# Priming of the autonomic nervous system after an experimental human pain model

**DOI:** 10.1152/jn.00064.2023

**Published:** 2023-07-05

**Authors:** Paulina Simonne Scheuren, Sofia Bösch, Jan Rosner, Florin Allmendinger, John Lawrence Kipling Kramer, Armin Curt, Michèle Hubli

**Affiliations:** ^1^Spinal Cord Injury Center, Balgrist University Hospital, University of Zurich, Zurich, Switzerland; ^2^Department of Neurology, University Hospital Bern, Inselspital, University of Bern, Bern, Switzerland; ^3^Danish Pain Research Centre, Department of Clinical Medicine, Aarhus University, Aarhus, Denmark; ^4^International Collaboration on Repair Discoveries (ICORD), University of British Columbia, Vancouver, British Columbia, Canada; ^5^Department of Anesthesiology, Pharmacology & Therapeutics, Faculty of Medicine, University of British Columbia, Vancouver, British Columbia, Canada

**Keywords:** autonomic nervous system, central sensitization, priming, secondary hyperalgesia, sympathetic skin response

## Abstract

Modulated autonomic responses to noxious stimulation have been reported in experimental and clinical pain. These effects are likely mediated by nociceptive sensitization, but may also, more simply reflect increased stimulus-associated arousal. To disentangle between sensitization- and arousal-mediated effects on autonomic responses to noxious input, we recorded sympathetic skin responses (SSRs) in response to 10 pinprick and heat stimuli before (PRE) and after (POST) an experimental heat pain model to induce secondary hyperalgesia (EXP) and a control model (CTRL) in 20 healthy females. Pinprick and heat stimuli were individually adapted for pain perception (4/10) across all assessments. Heart rate, heart rate variability, and skin conductance level (SCL) were assessed before, during, and after the experimental heat pain model. Both pinprick- and heat-induced SSRs habituated from PRE to POST in CTRL, but not EXP (*P* = 0.033). Background SCL (during stimuli application) was heightened in EXP compared with CTRL condition during pinprick and heat stimuli (*P* = 0.009). Our findings indicate that enhanced SSRs after an experimental pain model are neither fully related to subjective pain, as SSRs dissociated from perceptual responses, nor to nociceptive sensitization, as SSRs were enhanced for both modalities. Our findings can, however, be explained by priming of the autonomic nervous system during the experimental pain model, which makes the autonomic nervous system more susceptible to noxious input. Taken together, autonomic readouts have the potential to objectively assess not only nociceptive sensitization but also priming of the autonomic nervous system, which may be involved in the generation of distinct clinical pain phenotypes.

**NEW & NOTEWORTHY** The facilitation of pain-induced sympathetic skin responses observed after experimentally induced central sensitization is unspecific to the stimulation modality and thereby unlikely solely driven by nociceptive sensitization. In addition, these enhanced pain-induced autonomic responses are also not related to higher stimulus-associated arousal, but rather a general priming of the autonomic nervous system. Hence, autonomic readouts may be able to detect generalized hyperexcitability in chronic pain, beyond the nociceptive system, which may contribute to clinical pain phenotypes.

## Graphical abstract

**Figure:**
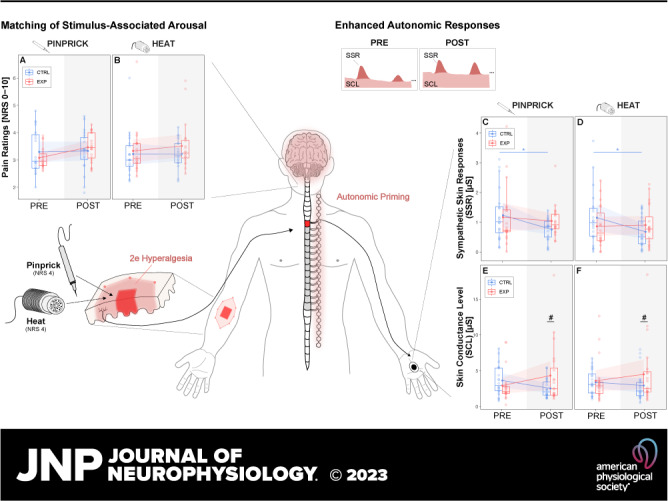

## INTRODUCTION

Pain is a multidimensional, subjective experience encompassing not only perceptual but also autonomic responses (1). The role of the autonomic nervous system in pain has been studied using a variety of measurement tools, such as electrodermal activity [e.g., skin conductance levels (SCL) and sympathetic skin responses (SSRs)], heart rate, heart rate variability, blood pressure, and pupil dilation (2–7). In individuals with chronic pain, autonomic responses to noxious stimuli are enhanced (e.g., increased SSRs) (8–12), paralleling that seen in healthy individuals after the experimental induction of secondary hyperalgesia (7). Emerging from these observations is the concept that autonomic readouts could serve as reliable and objective markers of nociceptive sensitization (7, 13, 14), discussed as one of the main potential underlying mechanisms of chronic pain (15, 16). Increased autonomic responses could, on the one hand, be directly attributable to sensitization of nociceptive and autonomic pathways. On the other hand, there is also the distinct possibility that sensitization in the nociceptive system results in more intense pain, which, in turn, drives greater arousal, ultimately modulating the autonomic nervous system. This is an important distinction because, only in the case of the former is the nociceptive system a viable target for analgesic interventions.

The aim of this study was to disentangle the role of nociceptive sensitization and stimulus-associated arousal on autonomic responses to noxious stimulation. This was achieved by way of comparing both pinprick- and heat-induced SSRs in the area of secondary hyperalgesia while controlling for stimulus-associated arousal by matching pain intensities of applied stimuli. Given the distinct psychophysical characterization of the area of secondary hyperalgesia (i.e., mechanical, but not heat hyperalgesia) (17), we hypothesized that SSRs will be enhanced in response to pinprick, but not to heat stimulation of the secondary hyperalgesia area. As such, the enhanced autonomic responses observed in the secondary area of hyperalgesia would be primarily mediated by nociceptive sensitization and not stimulus-associated arousal.

## MATERIALS AND METHODS

### Individuals

Twenty healthy females between the ages of 18 and 40 yr were recruited for the study. Exclusion criteria included any neurological disease, sensory deficits, acute or chronic pain, intake of any analgesic medication within 24 h before the study, regular intake of antidepressants, opioids, benzodiazepines, or anticonvulsants. The study was approved by the local ethics board “Kantonale Ethikkommission Zürich” (Reference Number: EK-04/2006, PB_2016-02051) and was in accordance with the Declaration of Helsinki. All individuals provided written informed consent. In addition to the exclusion criteria listed above, all individuals had to refrain from any physical exercise and the intake of alcohol, nicotine, of caffeine on the day of testing (i.e., at least 12 h before testing) as these can have a significant effect on autonomic readouts (18).

### Study Design

All individuals participated in two study visits (described in the next paragraph). Before all testing, all individuals had to complete a medical history questionnaire to assess any exclusion criteria not detected upon recruitment. Moreover, a semiquantitative sensory examination of the volar forearm was performed in all individuals to exclude the presence of subclinical sensory alterations. This included testing of light touch sensation using a cotton swab, discrimination between dull and pricky sensations using a safety pin, and cold and warm thermoroller testing (SenseLab Rolltemp II, Somedic SenseLab AB, Sweden). After sensory testing, individuals were asked to fill out the Pain Catastrophizing Scale (PCS) (19) and the Hospital Anxiety and Depression Scale (HADS) (20) to assess catastrophic thinking related to painful experiences as well as levels of anxiety and depression. Fatigue [numeric rating scale (NRS) 0 “no fatigue” to 4 “extreme fatigue”], physical activity, alcohol (amount of 0.3-L glasses), caffeine (number of cups), and nicotine (number of cigarettes) consumption in the timeframe of 24 h until 12 h before testing were assessed for each individual.

**Figure 1.**
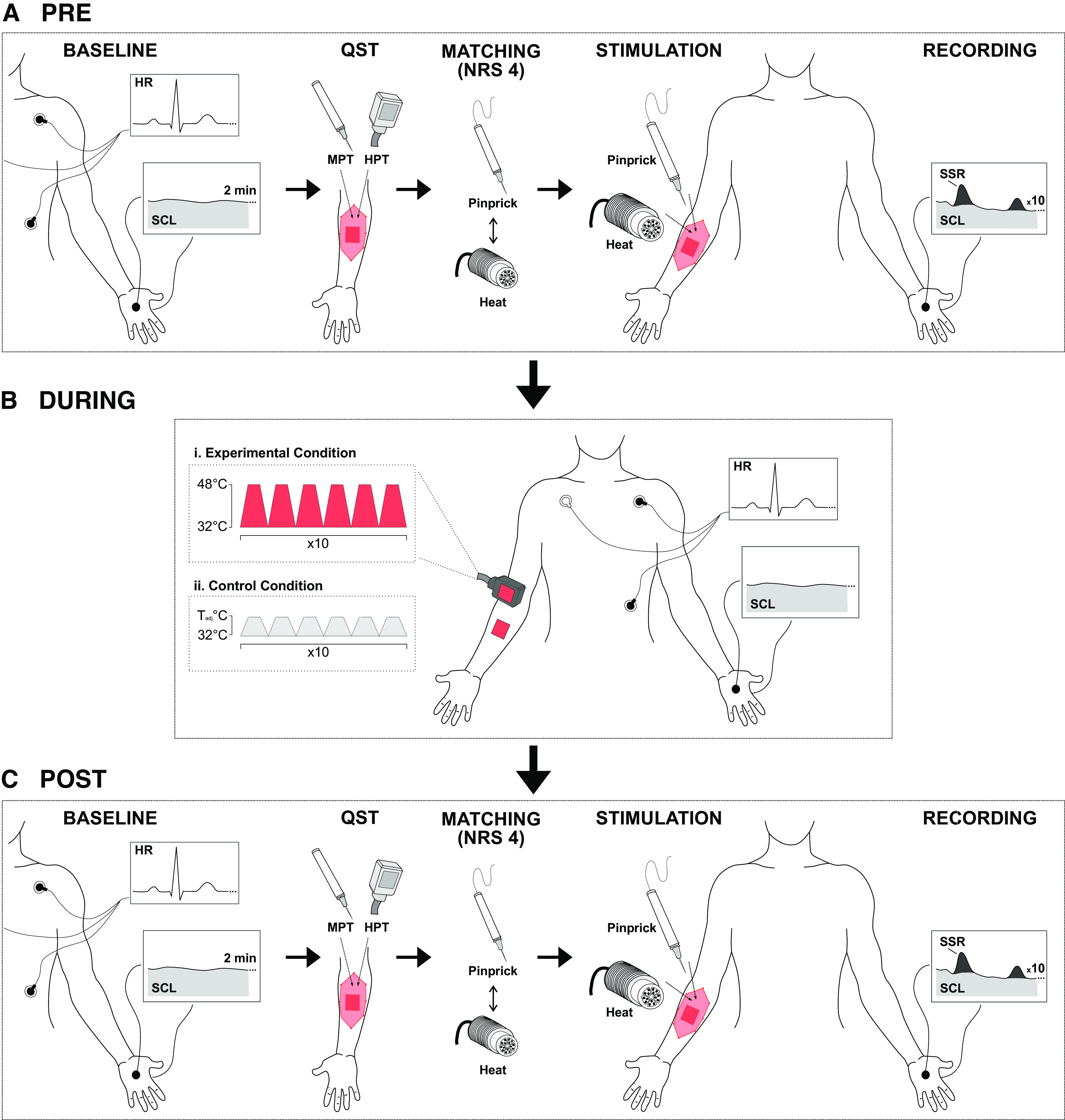
Experimental study design. The PRE-assessment (*A*) consisted of a 2-min baseline measurement of heart rate (HR) and skin conductance level (SCL) and quantitative sensory testing (QST) including mechanical (MPT) and heat pain thresholds (HPT). Then, all individuals underwent the matching paradigm [numeric rating scale (NRS) 4] for both pinprick and heat stimuli. Afterward, 10 noxious heat and 10 pinprick stimuli (randomized order) were applied to the proximal volar forearm with simultaneous recordings of sympathetic skin responses (SSRs) and SCL from the contralateral hand palm. During the application of the heat pain model (*Bi*) or control model (*Bii*), HR and SCL were assessed in both the experimental (*Bi*) and control (*Bii*) condition. Each stimulation block lasted ∼54 s (6 stimuli) with a 30-s interblock interval (total time was 13.5 min). The POST-assessment (*C*) included the same measurements as in the PRE-assessment and was performed in the area of secondary hyperalgesia (highlighted in light red). *T*_adj_, individually adjusted temperature of 1°C above the warm detection threshold.

Two study visits, i.e., an experimental (EXP) and control (CTRL) condition, were planned in a randomized order and separated by 2 wk. Each visit consisted of two time points (PRE and POST). All measures were performed before (PRE, Fig. 1*A*) and 20 min after (POST, Fig. 1*C*) a repetitive heat pain model in the EXP condition and a control model in the CTRL condition (DURING, Fig. 1*B*). Each time point (PRE and POST) for each condition (EXP and CTRL) consisted of a 2-min baseline measurement, a subset of the standardized quantitative sensory testing (QST), and a series of repetitive pinprick and noxious heat stimulation. All stimuli were applied to the volar forearm. The testing side (right/left arm) and the modality of the stimuli applied first (pinprick/heat) were randomized across all individuals. All individuals were asked to report the day postmenstruation at each study visit. The menstruation phase of their menstrual cycle was then defined as either follicular phase (cycle days 1–14) or luteal (cycle days 15–28).

### Quantitative Sensory Testing

Mechanical and heat pain thresholds were performed with standardized equipment and instructions provided by the German Research Network on Neuropathic Pain (DFNS) (21). The mechanical pain threshold was measured with commercially available weighted pinprick stimulators (8–512 mN; MRC Systems, Germany). The heat pain threshold (HPT) was assessed with the PATHWAY Pain and Sensory Evaluation System (Medoc Ltd., Ramat Yishai, Israel) Advanced Thermal Stimulator thermode (30 × 30 mm). A familiarization procedure was conducted before actual testing on the contralateral arm. All stimuli were applied outside of the marked area where the Advanced Thermal Stimulator thermode was applied for the repetitive heat pain model. In the EXP condition, this area corresponded to the area of secondary hyperalgesia after sensitization.

### Experimentally Induced Central Sensitization

A repetitive phasic heat pain model was used to induce secondary hyperalgesia. For this, noxious heat stimuli were applied to the center of the volar forearm with the 30 × 30 mm ATS thermode to induce primary and secondary hyperalgesia in the EXP condition (22). The paradigm consisted of 10 blocks of six noxious heat stimuli [baseline: 32°C; target: 48°C; duration 6 s; ramp 10 °C/s; no interstimulus interval (ISI): 0 s; interblock interval: 30 s] (Fig. 1*B*), resulting in a total heat pain model duration of 13.5 min (i.e., 54 s/block). All individuals were instructed to rate the intensity of each block on a NRS from 0 (“no pain”) to 10 (“most intense pain imaginable”). In the CTRL condition, the target temperature was adapted to 1°C above the warm detection threshold of each individual, which was also assessed using the same PATHWAY Pain and Sensory Evaluation System (Fig. 1*B*). After a 20-min break, the area of secondary hyperalgesia was mapped using a calibrated 256 mN von Frey filament (Optihair2, Marstock Nervtest, Germany) (22). VonFrey stimuli were first applied ∼10–15 cm outside of the hyperalgesic area and displaced toward the center (primary area of hyperalgesia) in 5-mm steps starting from eight different predefined angles. With their eyes closed, all individuals were instructed to indicate the point at which they felt a clear change in sensation from touch to pain. This location was marked on the skin for the eight different angles. The area of primary and secondary hyperalgesia was transferred to a transparent sheet and scanned for further analysis (22).

### Pinprick and Heat Stimulation

The pain intensity of all pinprick and heat stimuli was individually adapted to an NRS 4 across all time points (PRE/POST) and conditions (EXP/CTRL). Starting with the 8-mN pinprick stimulator, the stimulus force (8–512 mN) was increased repeatedly until a given force was consistently rated with the desired pain intensity (NRS 4) at least three times. This pinprick force was then used for the following testing session. Heat stimuli (baseline: 35°C, target: 60°C; ramp: 200 °C/s; 3 × 3.2 × 2.4 mm) were applied with a Thermal Cutaneous Stimulator 1.2 cm^2^ thermode (T03, QST.Lab, France). As the stimulus intensity was already set to maximal (60°C), we continuously adapted the stimulus duration (150–600 ms) until the stimulus was consistently rated with the desired pain intensity (NRS 4) at least three times. This heat stimulus duration was then used for the following testing session. The heat and pinprick stimuli were individually adapted prior to each time point (PRE/POST) for each condition (EXP/CTRL).

In each stimulation series, 10 stimuli (ISI of 13–17 s) were applied outside of the marked area of the Advanced Thermal Stimulator thermode used for the EXP/CTRL model, which corresponded to the area of secondary hyperalgesia (in the EXP condition). If a flare response spread beyond the marked area of the Advanced Thermal Stimulator thermode (i.e., area of primary hyperalgesia), all stimuli were applied outside of this flare area, but still within the area of secondary hyperalgesia. Nine seconds after each stimulus, individuals were cued by an auditory tone to rate the perceived pain intensity on an NRS (0–10). A familiarization procedure was performed before actual testing on the contralateral arm. The pinprick stimulator was repositioned by 1 cm after each stimulus to avoid sensitization of primary afferents. As the total surface of the Thermal Cutaneous Stimulator thermode consists of five zones (3 × 3.2 × 2.4 mm for each zone) that can be activated independently in a randomized fashion, the thermode could remain in the same position throughout the 10 stimuli without sensitizing primary afferents. Leaving the thermode in the same position was also important to avoid any additional mechanical stimulation of skin during thermal testing.

### Recording and Preprocessing of Autonomic Responses

**Figure 2.**
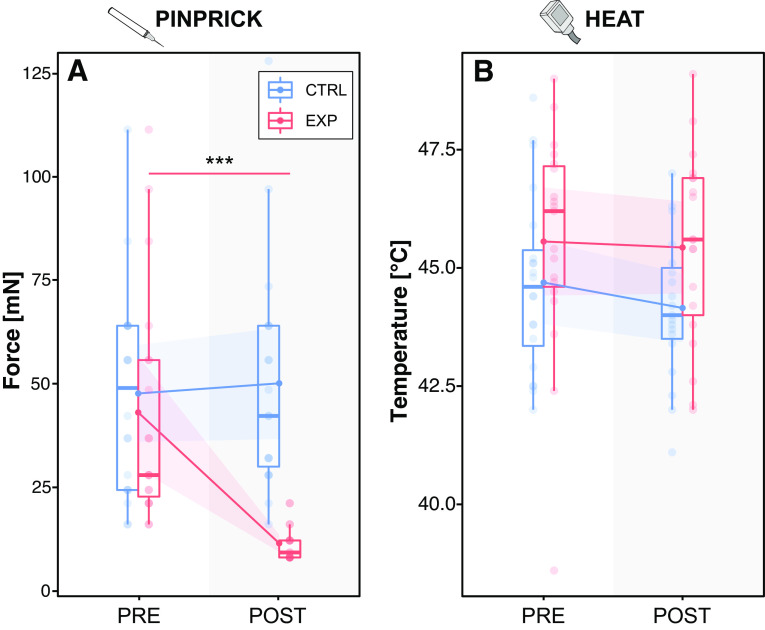
Quantitative sensory testing. The mechanical pain threshold (*A*) decreased from PRE to POST in the experimental (EXP, red) condition, but not in the control (CTRL, blue) condition in the secondary area. The heat pain threshold (*B*) did not differ between PRE and POST in both the EXP and CTRL condition in the secondary area. Data is shown for all individuals (*n* = 20). ***P < 0.001.

**Figure 3.**
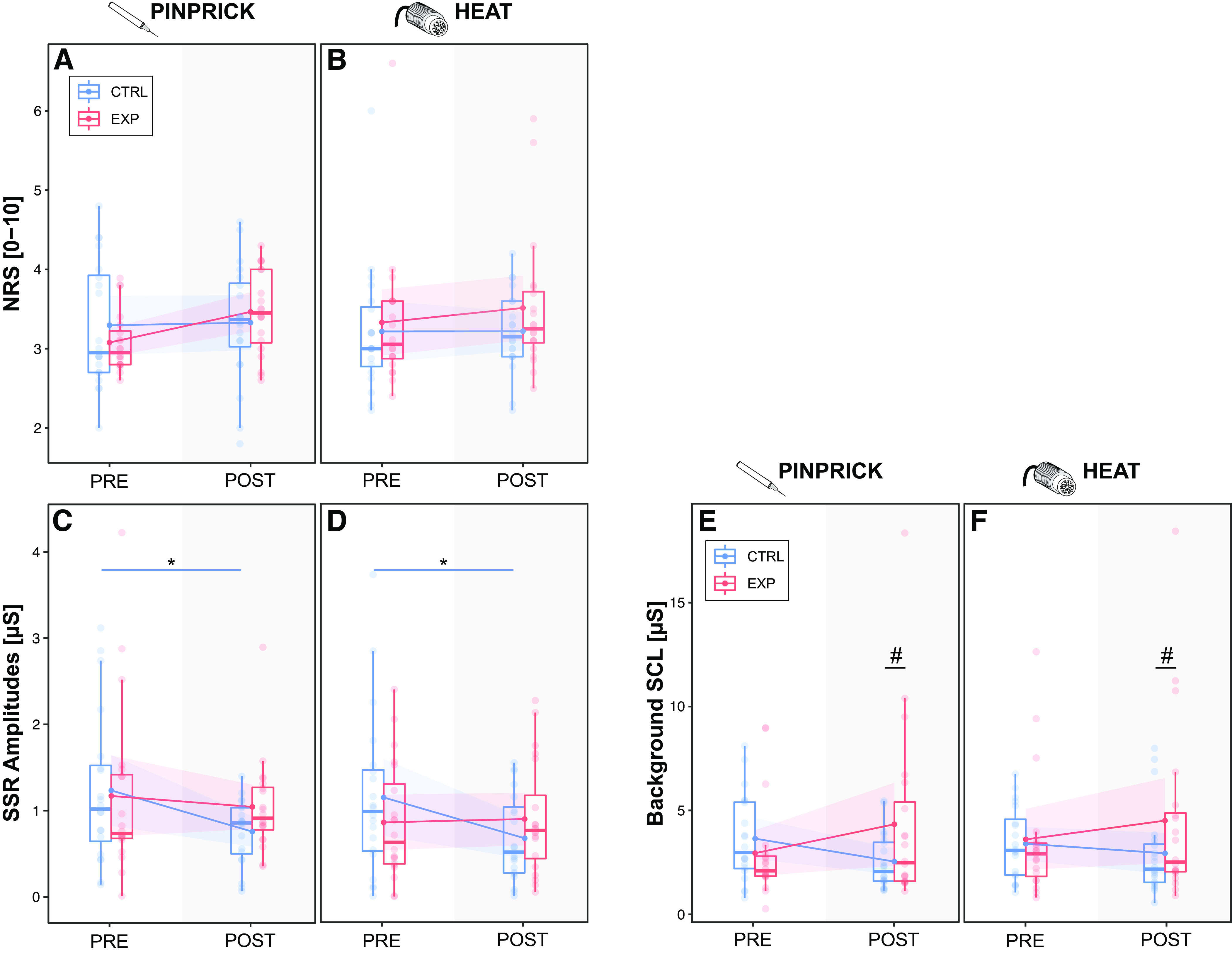
Pain ratings, sympathetic skin responses (SSRs), and background skin conductance (SCL). Pinprick-pain ratings (*A*), heat pain ratings (*B*), pinprick-induced SSRs (*C*), heat-induced SSRs (*D*), and SCL for pinprick (*E*) and heat (*F*) stimulation are shown for each timepoint (PRE/POST), condition (EXP/CTRL), and modality (pinprick/heat). Significances are shown for differences between timepoints (*P < 0.05) and conditions (*#P <* 0.05).

All autonomic responses were recorded with the data acquisition software LabChart (v.8.1.13, ADInstruments, Dunedin, New Zealand) and the corresponding data acquisition hardware PowerLab (ADInstruments, Dunedin, New Zealand). Electrodermal activity (i.e., SSRs and SCLs) was recorded from the hand contralateral to the testing side using Ag/AgCl electrodes (MLA0118 GSR Electrodes, ADInstruments, New Zealand) filled with a skin conductance electrode paste (MLA1095 Electrode Paste, ADInstruments, New Zealand). The skin was prepared with sandpaper tape (Red DotTM Trace Prep, 3M) and alcohol (Softasept N, B. Braun Medical AG, Germany). The active electrode was attached to the hand palm and the reference electrode to the dorsum of the hand. Skin temperature was assessed at the recording site and if the temperature was below 32°C, the skin was warmed with heating lamps. The electrodermal signal was amplified using a galvanic skin response amplifier (FE116 GSRamp, ADInstruments, New Zealand). Each stimulus (pinprick/heat) generated an automatic trigger signal. Electrodermal activity was analyzed in both *1*) phasic responses (SSRs) and *2*) tonic background skin conductance activity (SCL). Phasic SSRs were analyzed in a 7-s post-trigger window. SSR latencies were defined as the first deflection point of the signal after each trigger and SSR amplitudes (i.e., peak-to-peak responses) were automatically detected using a customized algorithm in MatLab (RRID:SCR_001622) and individually inspected to ensure correct signal detection. Signals contaminated with movement artifacts or nontime-locked responses were excluded offline. For the analysis of background tonic SCL, we used an open-source Matlab-based software (Ledalab). Continuous decomposition analysis of SC data into continuous signals of tonic and phasic activity was performed to separate continuous tonic (background SCL) from phasic data (SSR). Then, we separated the tonic SCL component into 10 sections (one section per stimulus), each within a 7-s post-trigger window. The average SCL for each of the 10 stimuli was computed. Baseline electrodermal activity was directly analyzed using the LabChart 8 Pro software during *1*) the 2-min PRE baseline (Fig. 1*A*), *2*) the first block of the heat pain model in the EXP condition and the control model in the CTRL condition (Fig. 1*B*), and *3*) the 2-min POST baseline (Fig. 1*C*).

An electrocardiogram was recorded using a 3-lead recording with disposable Ag/AgCl electrodes with a conductive adhesive hydrogel (Covidien plc, Ireland) placed below the left and right clavicles on the chest wall equidistant from the heart and below the left rib cage using the PowerLab acquisition system (PowerLab; ADInstruments, Dunedin, New Zealand). The recording sites were prepared with a skin preparation gel (Nuprep, D.O. Weaver & Co., Aurora, CO) and alcohol (Softasept N, B. Braun Medical AG, Germany). The ECG signal was band-pass filtered between 0.3 and 1,000 Hz and sampled at 1,000 Hz with the Dual Bio Amp (FE232, ADInstruments, Dunedin, New Zealand). To assess changes in adrenergic cardiovascular activity in addition to cholinergic sudomotor activity (i.e., SCL), heart rate and heart rate variability were assessed during *1*) the 2-min PRE baseline (Fig. 1*A*), *2*) the first block of the heat pain model in the EXP condition and the control model in the CTRL condition (Fig. 1*B*), and *3*) the 2-min POST baseline (Fig. 1*C*). Heart rate was assessed as an index of both sympathetic and parasympathetic activity, whereas heart rate variability [i.e., root mean square differences (rMSSD)] was used as an index of parasympathetic activity. Heart rate variability was analyzed online using the Heart Rate Variability Software Module (Labchart 8 Pro), which automatically detects beats by finding the R wave in the electrocardiogram signal. Beats affected by movement artifacts were removed offline before analysis.

### Data Analysis and Statistics

All statistical analyses were performed using R statistical software (v.4.1.0. mac OS Mojave 10.14.6). Statistical testing was performed according to data distribution, which was tested by means of histograms and quantile-quantile plots. The statistical significance was set at 0.05. Tukey’s adjustment was performed to adjust for multiple comparisons. Comparison of questionnaire data (i.e., tiredness, physical activity, and the consumption of alcohol, caffeine, and nicotine) between both study visits was done with a Wilcoxon signed-rank test.

General linear mixed models (function lmer from R package “lme4”) were used to assess the effect of condition (CTRL/EXP), modality (pinprick/heat), and time point (PRE/POST) on *1*) mechanical and heat pain thresholds, *2*) stimulus parameters (i.e., pinprick stimulus intensity/heat stimulus duration), *3*) pain ratings (pinprick/heat pain ratings), *4*) SSR amplitudes (pinprick- and heat-induced SSRs), and *5*) background SCL during both pinprick and heat stimulation. We used “individual” as a random effect (interaction condition × time point × modality). Post hoc multiple comparisons (R package “emmeans”) were performed between all possible combinations of condition (CTRL/EXP), modality (pinprick/heat), and time point (PRE/POST). Supplementary analyses were performed to assess the effect of the menstrual cycle on pain ratings, SSR amplitudes, and background SCL using general linear mixed models (condition + time + modality + menstrual cycle).

Spearman correlation analyses were performed to assess the relationship between SSR amplitudes and background SCL for both pinprick and heat stimulation after the experimental pain model (POST-assessment) (2, 4).

Heart rate, heart rate variability (rMSSD), and tonic baseline SCL were compared from PRE to DURING to POST in both the EXP and CTRL conditions. General linear mixed models were used to test the effect of time point (PRE/DURING/POST) and condition (CTRL/EXP) on *1*) heart rate, *2*) heart rate variability, and *3*) SCL with “individual” as a random effect. Post hoc multiple comparisons (“emmeans”) were performed between all possible combinations.

Finally, Spearman correlation analyses were performed to test the association between the degree of central sensitization (i.e., size of the area of secondary hyperalgesia), SSR amplitudes in the POST assessments, as well as heart rate, heart rate variability, and SCL during the pain model.

## RESULTS

### Individuals

Twenty healthy females (age 23.4 ± 3.1 yr) completed both study visits (CTRL and EXP conditions), separated by 15.9 ± 3.7 days. In the CTRL session, eight individuals reported being in the luteal phase of their menstrual cycle and 10 in the follicular phase. In the EXP session, 10 individuals reported being in the luteal phase of their menstrual cycle and eight reported being in the follicular phase of their menstrual cycle. Four individuals were in the same phase (luteal: *n* = 2; follicular: *n* = 2) at both the CTRL and EXP sessions. Two individuals were not able to report the timing of their menstrual cycle on both testing days (irregular/absent). All individuals presented with normal PCS (17.6 ± 7.4) and HADS (anxiety: 6.2 ± 2.9; depression: 2.7 ± 2.5) scores, except for one individual with a heightened HADS anxiety score of 14. There was no difference between CTRL and EXP conditions in terms of individual’s tiredness (CTRL: 1.2 ± 0.7; EXP: 0.9 ± 0.7, *W* = 22.5, *P* = 0.179), physical activity (CTRL: 19.5 ± 32.7; EXP: 19.5 ± 35.5 min; *W* = 27.5, *P* = 1.000), alcohol consumption (CTRL: 0.3 ± 0.5; EXP: 0.4 ± 0.5 glasses; *W* = 14, *P* = 0.484), caffeine intake (CTRL: 0.4 ± 0.7; EXP: 0.2 ± 0.4 cups; *W* = 1.5; *P* = 0.586), and nicotine consumption (CTRL: 0.5 ± 1.2; EXP: 0.4 ± 1.2 cigarettes; *W* = 0.0, *P* = 1.000).

### Experimentally Induced Secondary Mechanical, but Not Heat Hyperalgesia

The heat pain model was perceived as painful (7.4 ± 1.6 NRS over all 10 blocks) by all individuals and led to a large area of secondary hyperalgesia (64.2 ± 18.3 cm^2^) in all individuals in line with that reported in previous studies (7, 22). The experimental pain model had a significant effect on *1*) the mechanical pain threshold (*F* = 18.458, *P* < 0.0001; interaction “condition × time”) and *2*) the pinprick stimulus intensity (leading to and NRS 4) (*F* = 548.050, *P* < 0.0001). In the EXP condition, the mechanical pain threshold decreased significantly from PRE (43.0 ± 28.3 mN) to POST (11.4 ± 4.3 mN) in the EXP condition (*t* = 5.704, *P* < 0.0001) demonstrating secondary hyperalgesia to pinprick stimuli (Fig. 2*A*). In addition, the intensity of the pinprick stimulus applied during testing (i.e., consistently rated as NRS 4) decreased significantly from PRE (499.2 ± 57.2 mN) to POST (108.8 ± 59.1 mN) in the EXP condition (*t* = 33.108, *P* < 0.0001), also indicating the development of secondary hyperalgesia to pinprick stimuli. In the CTRL condition, the mechanical pain threshold did not change from PRE (47.6 ± 25.0 mN) to POST (50.1 ± 27.9 mN) (*t* = −0.347, *P* = 0.730) (i.e., no secondary pinprick hyperalgesia). Moreover, the pinprick intensity did not differ between the PRE (499.2 ± 57.2 mN) and POST (499.2 ± 57.2 mN) assessments in the CTRL condition (i.e., no secondary pinprick hyperalgesia) (*t* = 0.000, *P* = 1.000).

The experimental pain model did not have an effect on *1*) the heat pain threshold (*F* = 0.217, *P* = 0.643; interaction “condition × time”) and *2*) the heat stimulus duration (leading to and NRS 4) (*F* = 0.000, *P* = 1.000). The heat pain threshold did not differ between the PRE and POST assessment for both the EXP (PRE: 45.6 ± 2.4°C; POST: 45.4 ± 2.0°C; *t* = 0.918; *P* = 0.363) and CTRL (PRE: 44.7 ± 1.9°C; POST: 44.2 ± 1.5°C; *t* = 0.254, *P* = 0.800) conditions (i.e., no secondary heat hyperalgesia) (Fig. 2*B*). In addition, the duration of the heat stimuli applied to induce SSRs (i.e., consistently rated as NRS 4) did not differ between the PRE and POST assessment for both the EXP (PRE: 388 ± 137 ms; POST: 388 ± 137 ms; *t* = 0.000, *P* = 1.000) and CTRL conditions (PRE: 380 ± 123 ms; POST: 380 ± 123 ms) (*t* = 0.000, *P* = 1.000).

### Subjective Pain Ratings during Pinprick and Heat Stimulation

All average pain ratings were successfully matched across all assessments and did not differ between modalities, timepoints, or conditions (Fig. 3, *A* and *B*). All model statistics can be found in Supplemental Tables S1*A* and S2*A*.

### Pinprick- and Heat-Induced Sympathetic Skin Responses and Background Skin Conductance

Albeit matched pain intensities, the experimental pain model had a significant effect on both pinprick- and heat-induced SSRs (“condition × time”: *F* = 4.628, *P* = 0.033). Post hoc comparisons revealed that pinprick-induced SSRs habituated from PRE to POST in the CTRL condition (PRE: 1.1 ± 1.4 µS, POST: 0.7 ± 0.7 µS; *t* = 2.358, *P* = 0.019), but not in the EXP condition (PRE: 0.9 ± 1.0 µS, POST = 0.9 ± 0.9 µS; *t* = −0.194, *P* = 0.847) (Fig. 3*C*). Heat-induced SSRs also habituated from PRE to POST in the CTRL condition (PRE: 1.2 ± 1.3 µS, POST: 0.7 ± 0.9 µS; *t* = 2.378, *P* = 0.019), but not in the EXP condition (PRE: 1.1 ± 1.2 µS, POST: 1.0 ± 0.8 µS; *t* = 0.628, *P* = 0.531) (Fig. 3*D*).

The experimental pain model also had a significant effect on background SCL (“condition × time”: *F* = 7.015, *P* = 0.009). Post hoc comparisons revealed increased background SCL in the POST-assessment in the EXP compared with CTRL condition during both pinprick (CTRL-POST: 2.5 ± 1.3 µS; EXP-POST: 4.3 ± 4.3 µS; *t* = −2.360, *P* = 0.019; Fig. 3*E*) and heat stimulation (CTRL-POST = 3.4 ± 1.7 µS; EXP-POST: 4.5 ± 4.4 µS; *t* = −2.064, *P* = 0.041; Fig. 3*F*). All model statistics and post hoc comparisons can be found in Supplemental Table S1, *B* and *C*, and Table S2, *B* and *C*.

The menstrual cycle did not have an effect on pain ratings or SSR amplitudes. There was a main effect of the menstrual cycle on background SCL, which was however abolished upon post hoc testing (Supplemental Tables S4.1 and S4.2).

**Figure 4.**
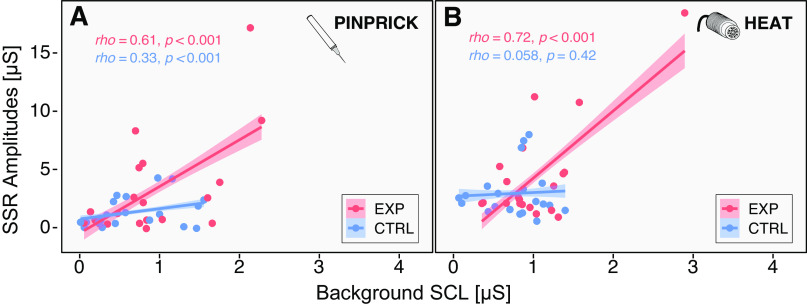
Correlation between background skin conductance level (SCL) and phasic sympathetic skin responses (SSRs). Spearman correlations between SCL and SSR in the POST-assessment are shown for both the pinprick (*A*) and heat (*B*) stimulation modality. The experimental (EXP) condition is shown in red and the control condition (CTRL) is shown in blue.

Interestingly, background SCL correlated positively with SSR amplitudes for both stimulus modalities (Fig. 4). Outliers were detected according to the interquartile range (IQR) [subtracting the first quartile (*Q*1) from the third quartile (*Q*3)] rule. Outliers were detected as any number greater than *Q*3 + (IQR × 1.5) or any number smaller than the *Q*1 – (1.5 × IQR). When removing the outliers, there was still a significant correlation between background SCL and SSRs (Supplemental Table S3).

### Heart Rate, Heart Rate Variability, and Tonic Skin Conductance Levels before, during, and after the Heat Pain Model

**Figure 5.**
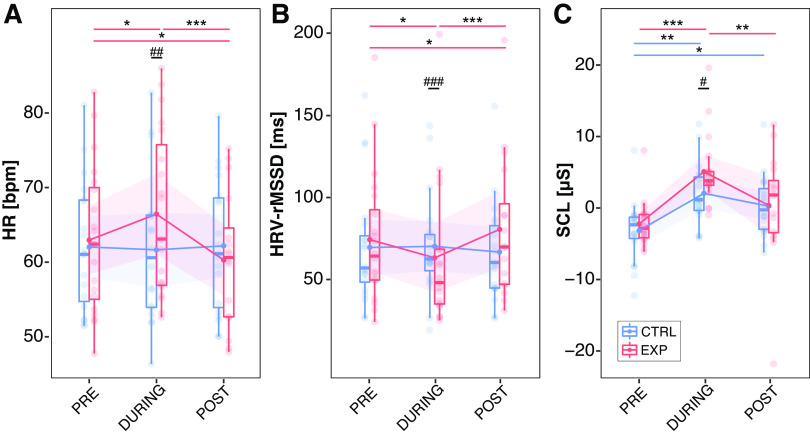
Changes in continuous autonomic readouts. Heart rate (HR, *A*), heart rate variability (HRV, *B*), and tonic skin conductance level (SCL, *C*) are shown at different timepoints (i.e., PRE, DURING, POST) in both the experimental (EXP, red) and control (CTRL, blue) condition. Significances are shown between timepoints (*P < 0.05; **P < 0.01; ***P < 0.001) and condition (#*P* < 0.05; ##*P* < 0.01; ###*P* < 0.001).

The heat pain model had a significant effect on heart rate (interaction “condition × time point”: *F* = 8.5, P < 0.001). In the EXP condition, heart rate increased from PRE (63.0 ± 9.8 beats/min) to DURING (66.5 ± 11.2 beats/min; *t* = −2.8, *P* = 0.02) and decreased from PRE to POST (60.3 ± 9.0 beats/min; *t* = 2.5, *P* = 0.04) and from DURING to POST (*t* = 5.1, *P* < 0.0001) (Fig. 5*A*). In the CTRL condition, heart rate did not change from PRE (62.5 ± 8.6 beats/min) to DURING (61.1 ± 10.0 beats/min; *t* = 1.2, *P* = 0.5) or POST (61.6 ± 9.0 beats/min; *t* = 0.7, *P* = 0.7). Heart rate also did not differ between the DURING and POST time points (*t* = −0.4, *P* = 0.9) in the CTRL condition. During the heat pain model (EXP), heart rate was higher compared with the CTRL condition (*t* = −4.1, *P* < 0.001). Heart rate did not differ between the EXP and CTRL conditions at the PRE (*t* = −0.3, *P* = 0.7) and POST (*t* = 1.4, *P* = 0.1) time points.

The heat pain model also had a significant effect on heart rate variability (interaction “condition × time point”: *F* = 3.2, *P* = 0.045). In the EXP condition, heart rate variability decreased from PRE (74.5 ± 40.2 ms) to DURING (63.4 ± 43.6 ms; *t* = −2.8, *P* = 0.02), increased from DURING to POST (80.7 ± 43.7 ms; *t* = 5.1, *P* < 0.0001) (Fig. 5*B*), and increased from PRE to POST (*t* = 2.5, *P* = 0.04). In the CTRL condition, heart rate variability did not differ from PRE (69.7 ± 36.0 ms) to DURING (70.4 ± 31.3 ms; *t* = 1.1, *P* = 0.5) and to POST (66.9 ± 30.8 ms; *t* = −0.4, *P* = 0.9). There was also no difference in heart rate variability between the DURING and POST time point (*t* = 0.7, *P* = 0.7)). During the heat pain model (EXP), heart rate variability was lower compared with the CTRL condition (*t* = −4.2, *P* = 0.0001). There was no difference in heart rate variability between the conditions at the PRE and (*t* = −0.3, *P* = 0.7) POST time point (*t* = 1.5, *P* = 0.1).

The heat pain model also had a significant effect on tonic baseline SCL (*F* = 20.344, *P* < 0.0001). In the EXP condition, SCL increased from PRE (−2.2 ± 3.1 μS) to DURING (5.1 ± 4.9 μS; *t* = −5.343, *P* < 0.0001), and decreased from DURING to POST (0.4 ± 7.7 μS; *t* = 3.271, *P* = 0.004) (Fig. 5*C*). SCL did not differ between the PRE and POST time points (*t* = −1.761, *P* = 0.189) in the EXP condition. In the CTRL condition, tonic SCL increased from PRE (−3.1 ± 4.2 μS) to DURING (2.1 ± 4.3 μS; *t* = −3.674, *P* = 0.001) and to POST (0.3 ± 4.2 μS; *t* = −2.459, *P* = 0.042). There was no difference in SCL between the DURING and POST time point (*t* = 1.250, *P* = 0.427). During the heat pain model (EXP), SCL was higher compared with the CTRL condition (*t* = −2.29, *P* = 0.025). There was no difference in SCL between the conditions at the PRE and (*t* = −0.659, *P* = 0.512) POST time point (*t* = −0.075, *P* = 0.940).

### Correlation between the Degree of Central Sensitization and Sympathetic/Parasympathetic Activity during and after the Pain Model

The size of the area of secondary hyperalgesia was neither related to SSR amplitudes in the POST assessment (pinprick: *R* = 0.190, *P* = 0.430; heat: *R* = 0.380, *P* = 0.100), nor to sympathetic or parasympathetic activity during the heat pain model, i.e., heart rate (*R* = 0.001, *P* = 0.990), heart rate variability (*R* = 0.150, *P* = 0.380), and SCL (*R* = 0.130, *P* = 0.430).

## DISCUSSION

The aim of the present study was to disentangle the roles of sensitization and stimulus-associated arousal in the modulation of autonomic responses to noxious input. As in previous studies (7, 11), habituation of SSR evoked by noxious stimuli was reduced after experimentally induced central sensitization. Interestingly, decreased SSR habituation was observed regardless of stimulus modality and despite intensity matching to a participant’s subjective perception. These findings collectively suggest that enhanced autonomic responses to pain are neither merely related to stimulus-associated arousal nor to sensitized nociceptive pathways, but rather linked to priming of the autonomic nervous system. The latter might render the autonomic nervous system more susceptible to incoming noxious stimuli, independent of the stimulus modality and perceived intensity.

### Autonomic Responses to Pain Do Not Reflect Stimulus-Associated Arousal

We previously demonstrated that pinprick-induced SSRs can be modulated in a state of sensitization (7, 11). In these instances, the modulation of SSRs may also be driven by increased stimulus-associated arousal, given that pain is perceived as more intense (5, 23–26). A crucial aspect of the current study is that pain intensities of applied stimuli were matched across time points (PRE/POST), conditions (control/experimental), and modalities (pinprick/heat), assuring the same level of subjective pain throughout all assessments. Interestingly, pinprick-induced SSRs habituated from PRE to POST in the control, but not in the experimental condition (i.e., reduced SSR habituation), indicating that the SSR amplitude is not solely related to the perceived subjective pain intensity. The present findings thus further corroborate our previous data (7) and negate a strict linear relationship between perceived subjective pain intensity and the magnitude of autonomic responses to the same stimulus. Enhanced pinprick-induced SSRs after the pain model are thus independent of stimulus-associated arousal and may be driven by increased responsiveness of nociceptive neurons within the central nervous system (i.e., central sensitization). This can be explained by the presence of structural and functional interactions between the nociceptive and autonomic nervous system, which occur at multiple levels of the neuraxis (27, 28). In other words, if the afferent nociceptive input becomes sensitized, this may in turn lead to increased efferent autonomic output, rendering the latter a potential objective readout of nociceptive sensitization (11, 12). Nevertheless, SSR amplitudes were not related to the area of secondary hyperalgesia after the pain model, indicating that autonomic responses may not be able to detect different degrees of sensitization.

### Dissociation between Psychophysical and Autonomic Readouts

In line with previous reports (29–31), the area of secondary hyperalgesia was marked by increased sensitivity to mechanical stimuli (i.e., reduced mechanical pain threshold in the POST-experimental condition, Fig. 2*A*), with the most prominent observation being the reduced variability of the mechanical pain threshold. This low variability might, to a certain degree, also be due to a floor effect of the applied pinprick force (8 mN). Preclinical studies have also confirmed this phenomenon, demonstrating increased firing of spinal nociceptive neurons to punctate mechanical, but not heat stimuli after experimentally induced secondary hyperalgesia (32–34). With this in mind, we hypothesized that SSRs would parallel psychophysical results and only expected modulation of pinprick-, but not heat-induced SSRs after the experimental pain model. Surprisingly, both pinprick- and heat-induced SSRs presented with a similar time course (i.e., pronounced physiological SSR habituation in the control condition, but reduced SSR habituation in the experimental condition). As the enhancement of heat-induced SSRs cannot be attributed to sensitization of nociceptive neurons within the spinal dorsal horn, which is known to be specific to only mechanical stimuli (32–34), alternative mechanisms of facilitation need to be considered.

Several cognitive factors may influence nociceptive processing and/or autonomic responses, such as attention (35–37) and expectation (38–41). In the present study, individuals were completely naïve as to the effects of the experimental pain model and were not instructed to expect more pain in the experimental condition. However, the experimental pain model was perceived as painful in all individuals (mean NRS = 7), which might still have led to higher expected pain in the POST assessments of the experimental- compared with the control condition. Another factor to consider is saliency (42–44), which makes a stimulus particularly noticeable (45–47) and has been shown to influence brain responses to both nociceptive and non-nociceptive stimuli (42, 48). Although both pinprick and heat stimuli were matched in terms of pain intensity, the heat stimuli may still have been perceived as more salient or unpleasant than the pinprick stimuli and thus have led to increased autonomic responses. The aforementioned factors may influence autonomic responses to noxious input and could account for the observed, yet unexpected modulation of heat-induced SSRs in experimental condition. Therefore, continued refinements in experimental paradigms should be sought to match saliency and/or unpleasantness across sensory modalities as well as thoroughly document expectation.

### Priming of the Autonomic Nervous System

An important question that arises is whether the modality-unspecific facilitation of SSRs in the area of secondary hyperalgesia is modulated by nociceptive or autonomic mechanisms. We observed a clear increase in cholinergic sudomotor activity (i.e., increased SCL) as well as changes in adrenergic cardiovascular activity (i.e., increased heart rate and decreased heart rate variability) during the heat pain model as compared with the CTRL condition. These changes were transient in nature and recovered after the heat pain model, demonstrating an expected physiological response of the autonomic nervous system to acute pain (3, 4, 39, 49, 50). In addition, these observed changes in heart rate, heart rate variability, and SCL did not correlate with the size of the area of secondary hyperalgesia, which indicates that the activation of the autonomic nervous system during the pain model is not necessarily related to the degree of sensitization. This is in line with previous studies demonstrating that experimentally induced hyperalgesia is not related to the degree of cutaneous sympathetic sudomotor activity (51, 52).

Although SCL recovered after the heat pain model when assessed at rest (2-min POST-baseline), the novel application of noxious stimuli, both pinprick and heat, led to a concomitant increase in background SCL (assessed during the application of phasic stimuli in the experimental condition), which also correlated with SSR amplitudes. This is an important distinction, as the increase in SSR amplitudes is not necessarily due to an overall increase in resting autonomic activity, but rather due to an increased responsiveness, which only becomes evident in the case of incoming noxious stimuli. This increased responsiveness of the autonomic nervous system to noxious heat and pinprick stimuli may be related to a state of priming, which was promoted by the repetitive heat pain model. The concept of priming has been well described for the nociceptive system (e.g., for postsurgical pain), demonstrating that latent pain sensitization may occur after surgery and may be important for the transition from acute to chronic pain (16, 53).

### Perspectives and Significance

The current findings indicate that enhanced autonomic responses to noxious stimuli after experimentally induced sensitization are not solely a consequence of stimulus-associated arousal, but rather due to priming of the autonomic nervous system. Therefore, this study further expands the utility of autonomic readouts to detect hypersensitivities in a variety of pain patients, as they may help to detect sensitization of the autonomic nervous system in patients with chronic pain. In particular, sensitization processes may occur beyond the nociceptive system and lead to a generalized state of hyperexcitability, including the autonomic nervous system. Such priming of the autonomic nervous system may heavily contribute to clinical pain phenotypes and, if assessed accurately, should therefore be considered as an essential target for pain management (44).

## DATA AVAILABILITY

The data that support the findings of this study are available from the corresponding author.

## SUPPLEMENTAL DATA

10.5281/zenodo.7962950 Supplemental Tables S1–S4: https://doi.org/10.5281/zenodo.7962950.

## GRANTS

The study was supported by the Clinical Research Priority Program of the University of Zurich (CRPP Pain) and the Swiss National Science Foundation Grant (32003B_200482). J.R. is supported by a postdoctoral fellowship grant by the International Foundation for Research in Paraplegia (P191F). J.L.K.K. is supported by a Natural Sciences and Engineering Research Council of Canada Discovery grant (RGPIN-2021-02866).

## DISCLOSURES

No conflicts of interest, financial or otherwise, are declared by the authors.

## AUTHOR CONTRIBUTIONS

P.S.S., J.R., A.C., and M.H. conceived and designed research; P.S.S. and S.B. performed experiments; P.S.S., S.B., and F.A. analyzed data; P.S.S., S.B., J.R., F.A., J.L.K.K., A.C., and M.H. interpreted results of experiments; P.S.S. prepared figures; P.S.S. and S.B. drafted manuscript; P.S.S., S.B., J.R., F.A., J.L.K.K., A.C. and M.H. edited and revised manuscript; P.S.S., S.B., J.R., F.A., J.L.K.K., A.C., and M.H. approved final version of manuscript.
